# Biofilm modelling on the contact lenses and comparison of the in vitro activities of multipurpose lens solutions and antibiotics

**DOI:** 10.7717/peerj.9419

**Published:** 2020-06-24

**Authors:** Sibel Dosler, Mayram Hacioglu, Fatima Nur Yilmaz, Ozlem Oyardi

**Affiliations:** Department of Pharmaceutical Microbiology, Istanbul University, Faculty of Pharmacy, Istanbul, Turkey

**Keywords:** Contact lenses, Biofilm modelling, In vitro activity, Multipurpose CL solutions

## Abstract

During the contact lens (CL) usage, microbial adhesion and biofilm formation are crucial threats for eye health due to the development of mature biofilms on CL surfaces associated with serious eye infections such as keratitis. For CL related eye infections, multi drug resistant *Pseudomonas aeruginosa* or *Staphylococcus aureus* (especially MRSA) and *Candida albicans* are the most common infectious bacteria and yeast, respectively. In this study, CL biofilm models were created by comparing them to reveal the differences on specific conditions. Then the anti-biofilm activities of some commercially available multipurpose CL solutions (MPSs) and antibiotic eye drops against mature biofilms of *S. aureus*, *P. aeruginosa*, and *C. albicans* standard and clinical strains were determined by the time killing curve (TKC) method at 6, 24 and 48 h. According to the biofilm formation models, the optimal biofilms occurred in a mixture of bovine serum albumin (20% v/v) and lysozyme (2 g/L) diluted in PBS at 37 °C for 24 h, without shaking. When we compared the CL types under the same conditions, the strongest biofilms according to their cell density, were formed on Pure Vision ≥ Softens 38 > Acuve 2 ∼ Softens Toric CLs. When we compared the used CLs with the new ones, a significant increase at the density of biofilms on the used CLs was observed. The most active MPS against *P. aeruginosa* and *S. aureus* biofilms at 24 h was Opti-Free followed by Bio-True and Renu according to the TKC analyses. In addition, the most active MPS against *C. albicans* was Renu followed by Opti-Free and Bio-True at 48 h. None of the MPSs showed 3 Log bactericidal/fungicidal activity, except for Opti-Free against *S. aureus* and *P. aeruginosa* biofilms during 6 h contact time. Moreover, all studied antibiotic eye drops were active against *S. aureus* and *P. aeruginosa* biofilms on CLs at 6 h and 24 h either directly or as 1/10 concentration, respectively. According to the results of the study, anti-biofilm activities of MPSs have changed depending on the chemical ingredients and contact times of MPSs, the type of infectious agent, and especially the CL type and usage time.

## Introduction

Contact lenses (CL) are used by over 230 million people worldwide for fixing the functional or optical eye problems, or for changing the appearance of the eyes (Morgan, Woods & Tranoudis, 2016). Under normal conditions, the corneal epithelial and stromal cells synthesize the innate defense factors such as chemokines, cytokines, antimicrobial peptides, proteins and surfactants that function as the major refractive structure for the eye (Kurpakus Wheater, Kernacki & Hazlett, 1999). When the CLs are inserted into the eyes, some substances such as proteins, glycoproteins, and lipids in the tear rapidly accumulate on CL surface, thus creating a suitable environment for microorganisms.

When the planktonic bacteria meet the CL surface, they first establish the microcolonies which is an early stage of the biofilm formation. Biofilm is a microbial community that can adhere to biotic or abiotic surfaces and produces extracellular polysaccharides. The microbial cells that grow in a biofilm are physiologically distinct from planktonic cells of the same organism and are more resistant to antibiotics, disinfectants or the host’s defense mechanisms. Development of mature biofilms on CL surfaces has been associated with keratitis in humans and animal models (Willcox, 2013; Bispo, Haas & Gilmore, 2015).

Keratitis which is a non-specific clinical term describing inflammation of the cornea, can cause a change in corneal structure and a decrease in its transparency. Corneal infectious agents include a wide range of pathogens such as bacteria, fungi and protozoa. Bacterial keratitis, especially CL-associated infections, are generally caused by both Gram-negative and Gram-positive pathogens such as *Pseudomonas aeruginosa* and *Staphylococcus aureus*, respectively. Fungal pathogens such as *Candida albicans* or *Fusarium* species are also very important, although their incidence is much lower than bacteria. Additionally, *Acanthamoeba* is a protozoon that causes a rare but aggressive form of infectious keratitis. Even though microbial keratitis is a rare complication of CL wear, it is one of the main causes of blindness in developing and developed countries (Dyavaiah, Phaniendra & Sudharshan, 2015; Evans & Fleiszig, 2013).

Disinfection plays an important role in CL related infections since the CLs can increase the risk of microbial adhesion and biofilm formation. Cleaning and disinfection of CLs are generally carried out by multipurpose CL solutions (MPSs). In addition, the existing efficient treatment is antibiotic eye drops for ophthalmological infections. There are various studies revealing the antimicrobial activities of MPSs or antibiotics against planktonic microorganisms. However, the number of these studies are not sufficient to evaluate the anti-biofilm activities of MPSs. For this purpose, we decided to test the anti-biofilm activities of MPSs, and antibiotic eye drops against biofilms on CLs.

Since the microbial adhesion rates on CL surfaces are sensitive to methodology and some environmental factors, it is necessary to choose and examine accurate and well-designed biofilms (Dutta, Cole & Willcox, 2012; Willcox, 2013). Therefore, in the first step of this study, CL biofilm models were created with common bacterial and fungal keratitis agents by comparing some differences in conditions. Then, we determined the anti-biofilm activities of some commercially available MPSs against *P. aeruginosa*, *S. aureus* and *C. albicans*; and antibiotic eye drops against *P. aeruginosa* and *S. aureus* standard and clinical strains’ mature biofilms on different kinds of new and used CLs by time killing curve analyses.

## Material and Methods

### Contact lenses

Four different types of CLs obtained commercially were used for biofilm models. The detailed information about CLs Pure Vision, Softens 38, Softens Toric and Acuve 2 were shown in Table 1. All four types of CLs used in the study were removed from their original packages and rinsed with sterile phosphate buffered saline (PBS) solution before using. CLs from FDA group I and group IV (Softens 38 and Acuve 2, respectively), which were routinely worn by the authors according to their ophthalmologist recommendations until the expiration date, were also included to the study for comparing.

**Table 1:** Characteristics of the contact lenses. FDA Group I: non-ionic, low water (<50% H_2_O); FDA Group 2: non-ionic, high water (>50% H_2_O); and FDA Group 4: ionic, high water (>50% H_2_O)).

	FDA group	Ion charge	Water content (%)	Diameter (mm)	Basic curve (mm)	United States adopted name (USAN)	Manufacturer
Pure Vision	I	Non-ionic	24	14.0	8.6	Lotrafilcon A	Bausch&Lomb
Softens 38	I	Non-ionic	38	14.0	8.7	Polymacon	Bausch&Lomb
Softens Toric	II	Non-ionic	64	14.5	8.5	Alphafilcon A	Bausch&Lomb
Acuve 2	IV	Ionic	58	14.0	8.7	Etafilcon A	Johnson&Johnson

**Notes.**

a Because the physicochemical properties of CLs from FDA Group 3 (ionic/low water) are similar to FDA Group 4, that CLs were not included in this study.

### Bacterial strains

Each two clinical isolates of *P. aeruginosa*, *S. aureus* and *C. albicans* were isolated from different patients in Clinical Microbiology Laboratories of Group Florence Nightingale Hospitals in Turkey. These isolates were identified with Vitek 2 system, and confirmed by routine biochemical tests and diagnostic API kits. *P. aeruginosa* ATCC 27853, *S. aureus* ATCC 43300 (methicillin resistant *S. aureus*/MRSA) and *C. albicans* ATCC 10231 (Rockville, MD, USA) were the standard strains used in the study.

### Media

Tryptic soy broth (TSB) and RPMI 1640 medium supplemented with 1% glucose were used for the cultures of bacteria and yeast, respectively. A mixture of bovine serum albumin (BSA) (20% v/v) and lysozyme (2 g/L) in PBS was used for the biofilm production assays, and tryptic soy agar (TSA) was used for colony counts.

### Antimicrobial substances

Four MPSs; All in One (Sauflon), Opti Free (Alcon), Bio True and Renu (Bausch&Lomb), and antibiotic eye drops; Genta (gentamycin 0,3%, I.E. Ulagay), Tobrased and Siprogut (tobramycin and ciprofloxacin 0,3% each, Bilim) were obtained commercially in the study. The contents of these four MPSs were shown in Table 2.

**Table 2:** The active ingredients of studied MPSs.

MPS	Active ingredients	Manufacturer
All in One Light	0,0001% poyheksanid 0,8% disodium phosphate dodekahydrate 1.0% poloksamer	Sauflon
Opti Free Express	0,001% polyquat (Polidronium Chloride) 0,0005% aldox (miristamidopropil dimetilamin)	Alcon
Renu Multiplus	0,03% hydroxyalkil phosphonate 1% polaksomine 0,0001% dymed (polyaminopropil biguanide)	Bausch & Lomb
Bio True	0.00013% polyaminopropyl biguanide 0.0001% polyquaternium	Bausch % Lomb

### Biofilm formation assays

CL biofilm models were created on four different types of new and used CLs by comparing the media, incubation time, temperature, and shaking. For this purpose, *S. aureus* and *P. aeruginosa* strains were cultured in five mL TSB whereas *C. albicans* strain was cultured in RPMI 1640 medium supplemented with 1% glucose, for 24 h at 37 °C with 360° rotation (50 rpm). These overnight cultures were diluted 1/10 in a glucose supplemented TSB /RPMI 1640 medium or in a mixture of BSA and lysozyme in PBS to act as the tear, yielding a final concentration of approximately 1×10^7^ cfu/ml. These suspensions (1 ml) were added to each CL types in a 24-well tissue culture microtiter plates. Plates were incubated at a temperature of either 37 °C or 25 °C, with or without shaking, for 24 h or 48 h. CLs were taken gently and washed three times with 1 ml PBS to remove unattached bacteria or yeast at the end of incubation period. CLs were shaken for five minutes with two mm sterile glass beads in order to decompose the biofilm. Following the disruption, serial 1/10-fold dilutions were performed and each 100 microliter sample was plated onto TSA. Following a 24 h incubation at 37 °C, colonies were counted. The negative controls were 1% glucose supplemented TSB /RPMI 1640 medium or a mixture of BSA and lysozyme in PBS. The experiments were repeated two times.

### Time killing curve (TKC) analyses

The modified TKC method, which we previously described, was used to determine the dynamic bactericidal or fungicidal activities of MPSs and bactericidal activities of antibiotics against biofilms on CLs (Dosler & Karaaslan, 2014). For this purpose, 24 h biofilms of standard and clinical *P. aeruginosa, S. aureus* and *C. albicans* strains on two types of new CLs (Softens 38 and Acuve 2), and 24 h mature biofilms of standard strains on used CL (Softens 38) were prepared with a mixture of BSA and lysozyme in PBS in 24-well tissue culture microtiter plates. MPSs or antibiotics were added to each corresponding well, and the plates were incubated for 0, 6, 24 and 48 h at 37 °C. After each incubation periods, CLs were washed two times with sterile PBS, and shaken with two mm sterile glass beads for five minutes. Following the disruption, serial 1/10-fold dilutions were carried out and 100 microliter samples were plated onto TSA. Colonies were counted after 24 h incubation at 37 °C. An antibiotic-free controls of each strain was also included to the tests. The experiments were repeated two times.

The lower detection limit was determined as 1 log_10_ cfu/mL, and bactericidal or fungicidal activity was defined as a ≥ 3-log_10_ cfu/mL decrease from the initial inoculum in TKC analyses (National Committee for Clinical Laboratory Standards, 1999).

### Fluorescent staining

Bacterial viability tests of standard *S. aureus*, *P. aeruginosa* and *C. albicans* biofilms on CLs before and after the treatment with Opti Free, the most active MPS, were performed with the L7007 LIVE/DEAD *Bac*light kit containing Syto-9 and propidium iodide dyes. The fluorescent reagents were diluted in saline and mixed at a 1:1 ratio. They were incubated for 15 min in the dark at 37 °C, and examined microscopically via Olympus CKX41. The green fluorescence was considered as living cells whereas red/orange was considered as non-living cells.

### Statistical analyses

In TKC analyses, results were presented as the mean ± standard deviations of two independent assays performed against standard and two clinical isolates. One-way analysis of variance (ANOVA) with Bonferroni’s multiple comparison tests was used to compare biofilm formations on different CL types. *P* values <0.001 were considered statistically significant.****

## Results

### Biofilm formation assays

When we created the different CL biofilm models on four different types of new and used CLs by comparing several conditions, we observed some similarities and some differences between these conditions. As shown in Figs. S1 and S2, while the media and temperature were affective factors for biofilm formation, shaking and incubation time (24 or 48 h) were not significantly affective. According to these results, we decided that the optimal biofilms occurred in a mixture of BSA and lysozyme in PBS at 37 °C for 24 h for bacteria and 48 h for yeast, without shaking.

**Figure 1:**
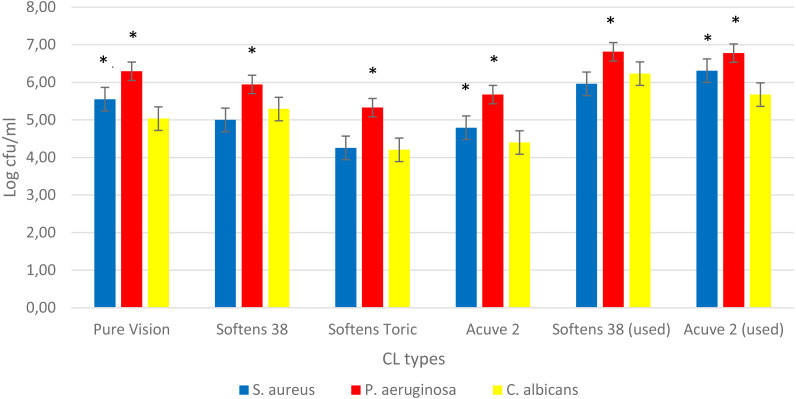
Comparison of the biofilms on CLs. Comparison of the biofilms formed on different CL types at 24 h for *S. aureus* (blue bars) and *P. aeruginosa* (red bars) and 48 h for *C. albicans* (yellow bars) standard strains. The X- and Y-axis represents CL types, and logarithmic microorganisms’ survival in biofilm, respectively. The asterisks indicate the statistical significance in biofilms density of microorganisms on CLs’ (P values < 0.001).

When four CL types were compared under the same conditions, as shown in Fig. 1, the strongest biofilms according to their cell density, were formed on Pure Vision ≥ Softens 38 > Acuve 2 ∼ Softens Toric. Although the strongest biofilm formed on Pure Vision, there was no statistically significant difference between the log cfu/ml numbers in biofilms on Softens 38 and Pure Vision. Similarly, there was no significant difference between biofilm capacities of Acuve 2 and Softens Toric. Then, due to the convenience of comparability with used CLs, we preferred to continue the study with Softens 38 and Acuve 2 as strong and semi-strong biofilm inducers, respectively.

When we compared the used CLs to new ones, we observed a significant increase at the density of biofilms on used CLs. Although, there was no significant difference between two types of used CLs, Softens 38 was more potent for biofilm. Thus we decided to continue with it to TKC analyses.

### Time killing curve (TKC) analyses

**Figure 2:**
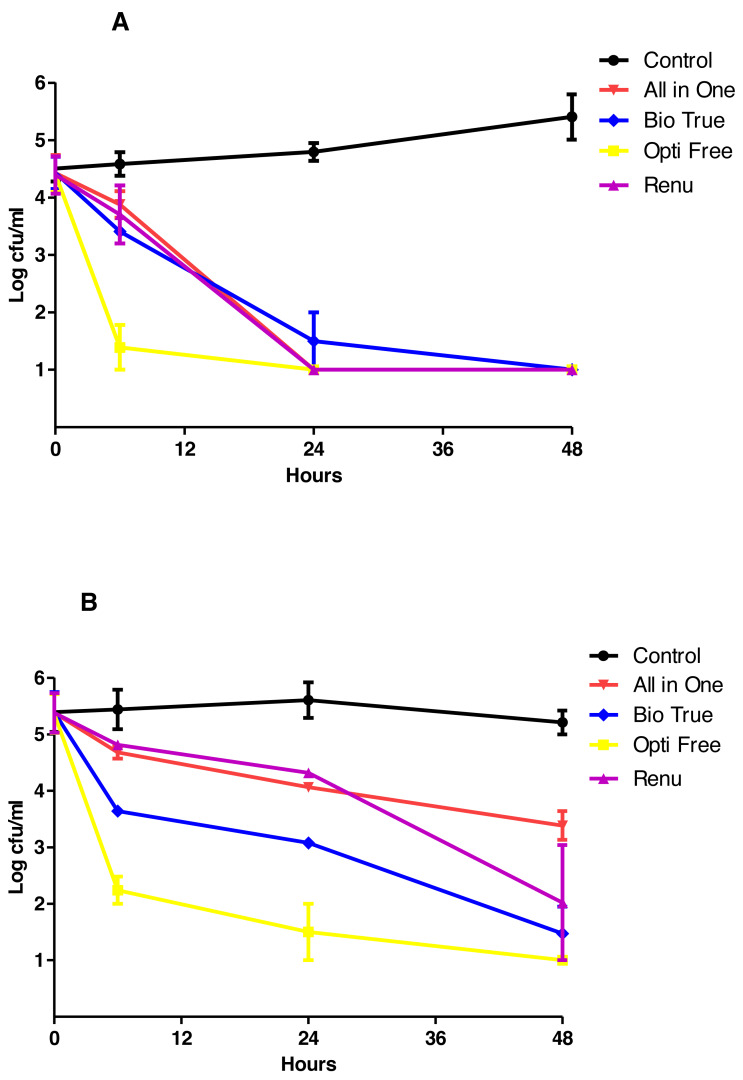
MPSs against *S. aureus* biofilms on CLs. The in vitro activities of MPSs observed by time-kill determinations against biofilms of standard and clinical *S. aureus* strains on (A) Softens 38 and (B) Acuve 2 CLs. The X- and Y-axis represents time, and logarithmic *S. aureus* survival in biofilm, respectively. Error bars indicate the standard deviations between data. cfu: colony-forming unit, Control: *S. aureus* biofilms without any antimicrobial treatment.

**Figure 3:**
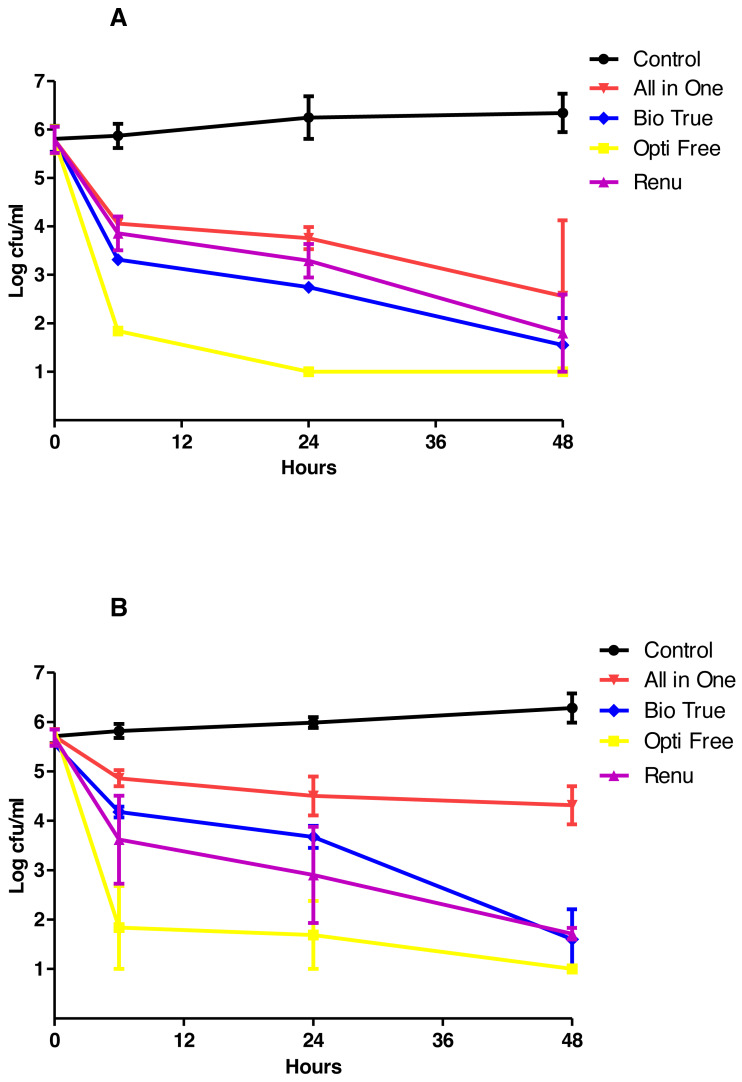
MPSs against *P. aeruginosa* biofilms on CLs. The in vitro activities of MPSs observed by time-kill determinations against biofilms of standard and clinical *P. aeruginosa* strains on (A) Softens 38, and (B) Acuve 2 CLs. The X- and Y-axis represents time, and logarithmic *P. aeruginosa* survival in biofilm, respectively. Error bars indicate the standard deviations between data. cfu: colony-forming unit, Control: *P. aeruginosa* biofilms without any antimicrobial treatment.

**Figure 4:**
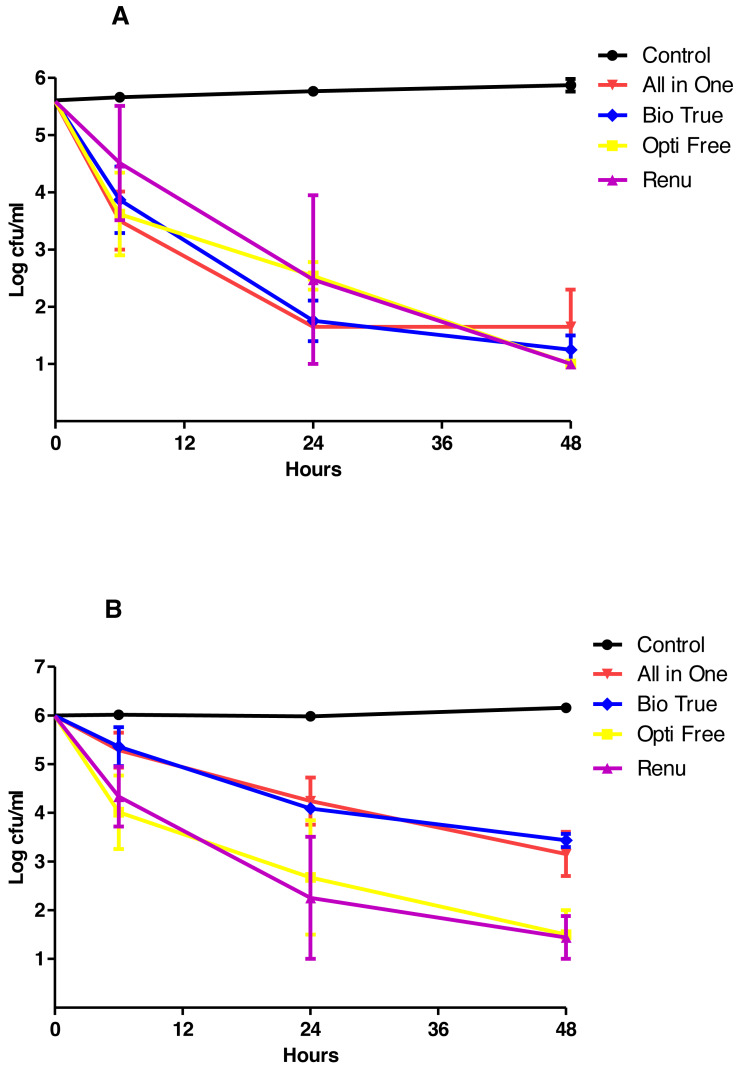
MPSs against *C. albicans* biofilms on CLs. The in vitro activities of MPSs observed by time-kill determinations against biofilms of standard and clinical *C. albicans* strains on (A) Softens 38, and (B) Acuve 2 CLs. The X- and Y-axis represents time, and logarithmic *C. albicans* survival in biofilm, respectively. Error bars indicate the standard deviations between data. cfu: colony-forming unit, Control: *C. albicans* biofilms without any antimicrobial treatment.

**Figure 5:**
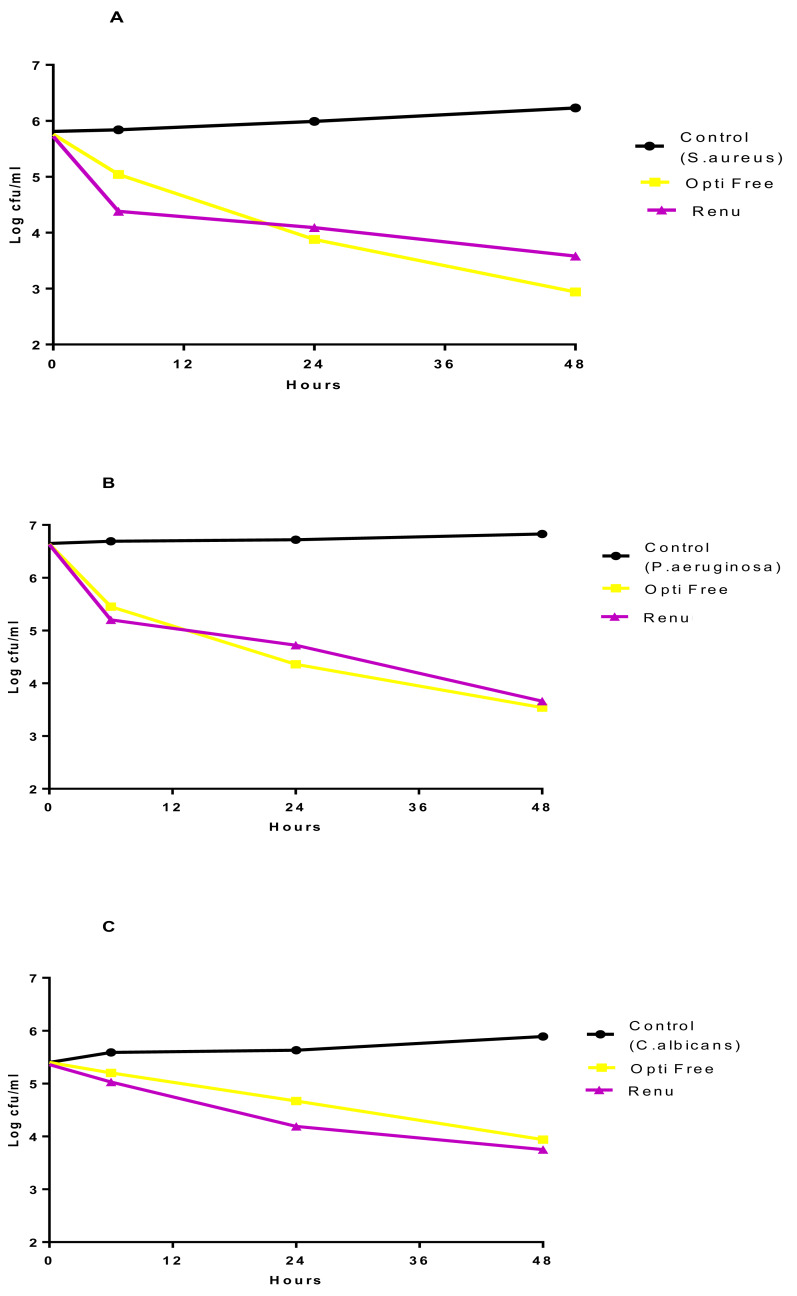
The effect of MPSs against biofilms on used CLs. Most active in vitro activities of MPSs observed by time-kill determinations against biofilms of standard strains (A) *S. aureus*, (B) *P. aeruginosa*, and (C) *C. albicans* on used CLs. The X- and Y-axis represents time, and logarithmic microorganisms’ survival in biofilm, respectively. cfu: colony-forming unit, Control: biofilms without any antimicrobial treatment.

The results of TKC analyses were shown in Figs. 2–4, and separate graphics for standard and clinical strains were shown in Figs. S3–S5. According to these results, the**** most active MPS against *P. aeruginosa* and *S. aureus* was Opti-Free followed by Bio-True and Renu at 24 h, and the most active MPS against *C. albicans* was Renu followed by Opti-Free and Bio-True at 48 h. In 6 h contact time, none of the MPSs showed 3-log_10_ cfu/mL bactericidal/fungicidal activity, except for Opti-Free against *S. aureus* and *P. aeruginosa* biofilms. When we performed the test on the used CLs, none of the MPSs reached 3-log_10_ cfu/mL bactericidal/fungicidal activity against the biofilms of standard strains at 24 h (Fig. 5).

**Figure 6:**
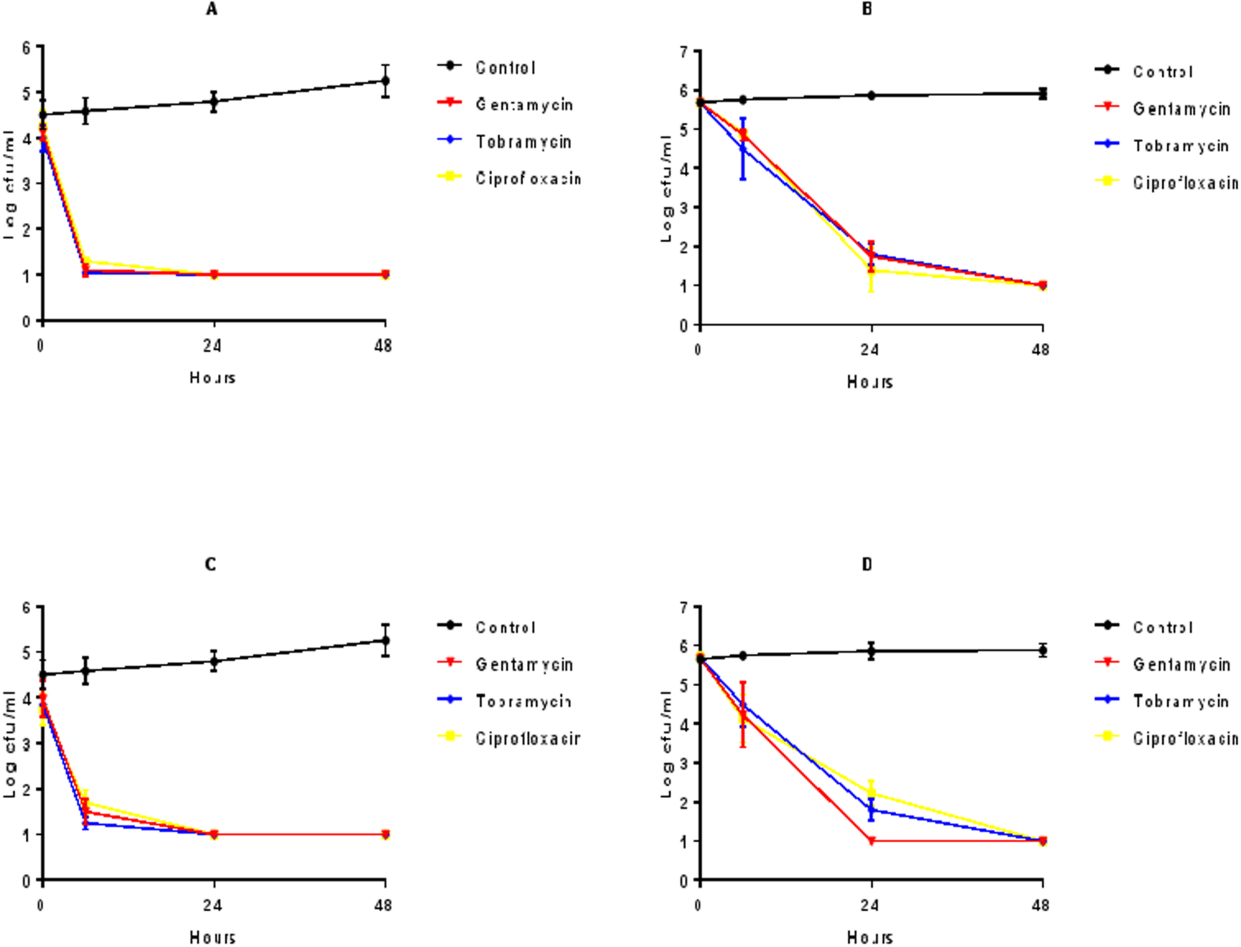
The effect of antibiotics against biofilms on CLs. Direct (A and C) and 1/10 diluted (B and D) antibiotic eye drops’ in vitro activities observed by time kill determinations against biofilms of standard and clinical *S. aureus* (A and B), and *P. aeruginosa* (C and D) strains on CLs. The X- and Y-axis represents time, and logarithmic *S. aureus* and *P. aeruginosa* survival in biofilm, respectively. Error bars indicate the standard deviations between data. cfu: colony-forming unit, Control: *S. aureus* and *P. aeruginosa* biofilms without any antimicrobial treatment.

As shown in Figs. 6A and 6C, all of the examined antibiotic eye drops gentamycin, tobramycin and ciprofloxacin showed ≥3-log_10_ cfu/mL bactericidal activity against *P. aeruginosa* and *S. aureus* biofilms on CLs at first 6 h and these activities continued for longer contact times when eye drops were used directly. Conversely, as shown in Figs. 6B and 6D, when we used them at 1/10 concentration for mimicking the approximate dilution rate on eye surface, they showed ≥3-log_10_ cfu/mL bactericidal activity at least 24 h.

### Fluorescent staining

**Figure 7:**
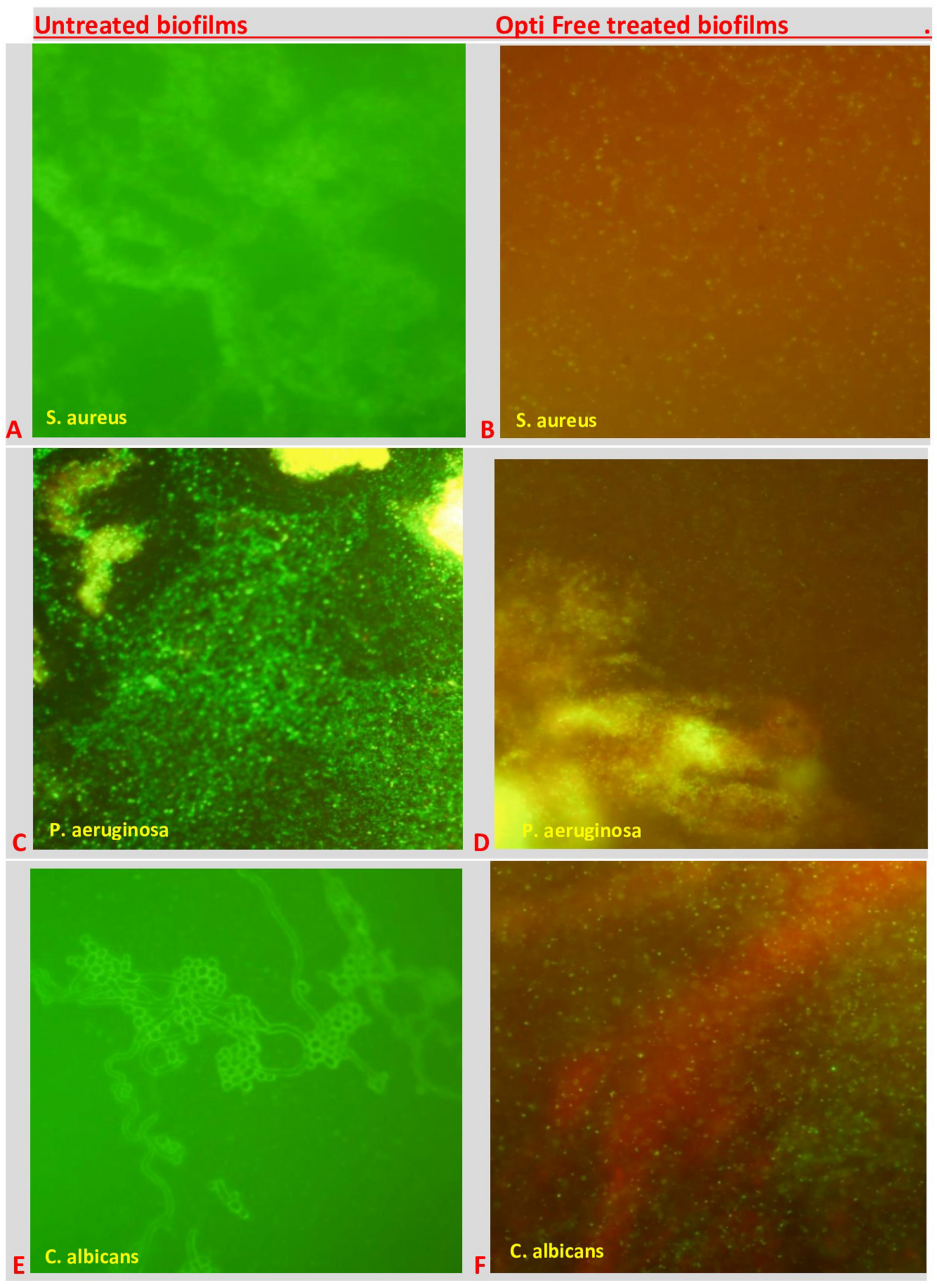
The visual effects of MPSs against biofilms on CLs. Untreated biofilms of (A) *S. aureus*, C: *P. aeruginosa*, and (E) *C. albicans*; and Opti Free treated biofilms of (B) *S. aureus*, (D) *P. aeruginosa*, and (F) *C. albicans*, where viable cells are stained green and dead cells are stained red.

When we performed the biofilm viability tests on CL surfaces by using the L7007 LIVE/DEAD Baclight kit, as shown in Fig. 7, we observed that there were living and non-living cells, before and after the treatment with Opti Free, respectively.

## Discussion

Microbial adhesion to CLs is believed to be an important cause of serious eye problems such as microbial keratitis, CL induced acute red eye, and peripheral ulcers during CL wearing period (Willcox & Holden, 2001; Sweeney et al., 2003). Although microbial keratitis has been known for many years as a corneal infection caused by low hygienic conditions, trauma or failure of the ocular surface, the most common risk factor today is CL wearing. Although CLs have brought some medical and aesthetic convenience to our lives, they also provide a new surface for biofilm forming pathogenic microorganisms in the eyes (Robertson, 2013; Ong & Corbett, 2015). In recent years, while the early diagnosis and effective antibiotic treatment of microbial keratitis are existing, new studies should be performed in order to understand and acquire more potent prevention and treatment methods.

As previously noted by several researchers, there are various factors affecting the bacterial adhesion on the CLs as the first step of biofilm formation (Dutta, Cole & Willcox, 2012). Therefore, successful and realistic biofilm studies are very important for the diagnosis and treatment of CL related eye infections. In this study, when we created the different CL biofilm models on different types of CLs; as shown in Fig. S1, the most important factor was the media, and then the temperature. As shown in Fig. S2, incubation time (24 or 48 h) was partially important for yeast but not for bacteria in biofilms on CLs. According to these results, we decided the optimal biofilms occurred in a mixture of BSA and lysozyme in PBS at 37 °C. These are the most similar conditions to the normal physiology in an eye wearing CL, and therefore our biofilm model was very accurate and realistic for CL related eye infections.

It is known that CL material plays an important role for microbial adhesion and could affect the frequency of CL related eye infections because the hydrophobicity of CL surfaces is distinctive for absorption of the tear film. In recent studies, daily disposable and silicone hydrogel CLs have been found more relevant for microbial keratitis than rigid gas permeable ones (Dart et al., 2008; Bruinsma, Van der Mei & Busscher, 2001). In addition, several studies about bacterial, fungal or protozoal (Acanthamoeba) biofilms on silicone hydrogel CLs have shown that water content of the biofilms also plays an important role for the biofilm capacities of CLs (Imamura et al., 2008; Dutta, Cole & Willcox, 2012; Reverey et al., 2014). In this study, as shown in Fig. 1, CL having more potent biofilm capacity has the lowest water content (24%), and more biofilm resistant CLs have the highest water contents (58% and 64%). Similar to other studies, these results suggest that increased water content of CLs makes them softer and easier in order to use for longer terms, and enables them more resistant to biofilm formation.

When we compared the biofilm capacities of used CLs with new ones, we observed a remarkable increase in the density of biofilms on the used CLs, regardless of their water content or ionic charge, as expected. This result has demonstrated that the corrosion occurred on the surface of used CLs, and this made them more sensitive to adhesion of microorganisms leading to the biofilm formation.

In this study, the differences between the most frequent eye pathogens’ biofilm capacities on CLs were also demonstrated. As shown in Fig. 1, both standard and clinical strains of *P. aeruginosa* were the most potent biofilm forming microorganisms on all kinds of CLs in every condition. Although the biofilm capacities of *S. aureus* and *C. albicans* were similar in the general sense, *S. aureus* biofilms were more potent on some CL types, especially on the used and unused Acuve 2 and Pure Vision. On the other hand, *C. albicans* biofilms were more potent than *S. aureus* only on the used and unused Softens 38.

Since the relationship between the biofilm formation on CLs and microbial infections has been shown, the disinfection process of CLs has become more important in order to prevent them from such infections (Al-Mujaini et al., 2009; Zimmerman, Nixon & Rueff, 2016). The process of CL cleaning with MPSs is very essential for removing the mucus, cosmetics and other residues from the surface and subsequent disinfection of them. The quality of MPSs is generally based on the guidelines of International Organization for Standardization (ISO) 14729 as “the solution should induce a 3 log reduction of reference bacterial or fungal strains” (International Standards Organization, 2001; Rosenthal, Sutton & Schlech, 2002). There are several studies examining the antimicrobial activities of MPSs against planktonic forms of the bacterial or fungal pathogens in the relevant literature. In these studies, the most active agent might be different in each study, but all MPSs have exhibited ≥3 log reduction from initial inoculum, confirming the required antimicrobial activity (Manuj et al., 2006; Mohammadinia et al., 2012; Kuzman et al., 2013; Kal, Toker & Kaya, 2017). Although ISO standards are suitable and effective for antimicrobial activities of MPSs against the planktonic forms of microorganisms, they are not sufficient for anti-biofilm activities.

When we tested the MPSs against biofilms on two types of CLs, the most active MPS against bacteria was Opti-Free, and against the yeast *C. albicans*, even though it was variable according to the strain and CL type, was Renu. While these differences were observed between bacteria and yeast, overall activities of MPSs did not significantly changed against the standard and clinical strains (Figs. S3–S5). Moreover, our fluorescence microscopy results clearly highlighted and confirmed the visual effects of MPSs against bacterial or fungal biofilms on CLs.

Similar to other studies, our results have shown that, there were major differences between anti-biofilm activities of MPSs (Szczotka-Flynn et al., 2009; Retuerto et al., 2012). We estimated that the most important factors were their chemical ingredients, contact time, and the target microorganism. When we considered the contact time, in first 6 h, none of the MPSs showed 3 Log bactericidal/fungicidal activity against biofilms on CLs, except for Opti-Free. These results indicated that either equal or less than 6 h contact time with MPSs was not sufficient to eradicate bacterial or fungal biofilms on CLs.

In this study, CL type was another significant factor affecting the anti-biofilm activities of MPSs. According to the results, although the biofilms on Softens 38 (38% water content, non-ionic) were stronger than the biofilms on Acuve 2 (58% water content, ionic), especially the *S. aureus* and *C. albicans* biofilms on Softens 38 could be eradicated considerably easier. Shoff et al. (2012) showed that the efficacy of MPSs was affected by some CL materials, especially etafilcon A and polyhexamethylene biguanide (poyheksanid). Similarly, in our study, MPS All in One showed decreased antimicrobial activity against all studied microorganisms’ biofilms on Acuve 2 CL.

On the other hand, when we tested the most active MPSs (Opti Free and Renu) on the used CLs, the activities decreased dramatically. As shown in Fig. 5, none of the MPSs achieved a 3 Log bactericidal/ fungicidal activity at 6 or 24 h, against any biofilms on the used CLs.

Similar to the studies on MPSs, there are various evidence-based analyses regarding antibacterial activities of antibiotic eye drops, but there is no evidence for their anti-biofilm activities (Teweldemedhin et al., 2017; Suzuki, Yamamoto & Ohashi, 2016). When we tested the antibiotic eye drops, all three agents were active against the bacterial biofilms on CLs for 6 h. But, when we diluted them to represent their eye concentrations, as shown in Fig. 6, the activities were decreased. These results suggested that the examined antibiotic eye drops in the study were effective in the treatment of CL related eye infections caused by *S. aureus* or *P. aeruginosa*. However, their contact time should be strictly considered, and their repeating doses should not be forgotten.

Despite the innovations in CL materials and MPSs ingredients, the occurrence rate and severity of microbial keratitis have not decreased significantly. Thus, there is a high interest towards engineering interventions for developing antimicrobial CLs and lens cases as prevention strategies such as using silver, free-radical producing agents, natural herbal compounds, antimicrobial peptides (AMP) or employing passive surface modification approaches (Sankaridurg, Lazondela Jara & Holden, 2013; El-Ganiny et al., 2017; Xiao et al., 2018). AMP coated CL is the most promising one among them, due to the AMPs excellent antibiofilm activities against mature or immature biofilms (Dosler & Karaaslan, 2014). Casciaro et al. (2018) have demonstrated that the frog skin AMP Esculentin-1a derived peptides represented encouraging candidates to be developed as ophthalmic formulations and/or for the manufacturing an antimicrobial CLs in order to prevent them from infections.

## Conclusion

Several factors play an important role in CL based biofilm studies. Thus, the optimum conditions should be provided to acquire optimal results. While the results of our study showed that the effective antibiotic therapy could be provided by antibiotic eye drops, the best choice is preventing the CL related eye infections with efficient disinfection of CLs by MPSs. In this study, the most active MPS against *S. aureus*, *P. aeruginosa* and *C. albicans* biofilms on CLs was Opti Free. In addition, the anti-biofilm activities of MPSs were based on various factors, such as chemical ingredients and contact time of MPSs, the type of infectious agent, and especially the CL type and usage time.

##  Supplemental Information

10.7717/peerj.9419/supp-1 Figure S1 Comparison of the some conditions for biofilm forming on CLs Comparison of the biofilms formed in different media and temperature conditions at 24 h for A: *S. aureus* and B: *P. aeruginosa* and 48 h for C: *C. albicans* standard strains. The X- and Y-axis represents CL types, and logarithmic microorganisms’ survival in biofilm, respectively.

10.7717/peerj.9419/supp-2 Figure S2 Comparison of shaking and incubation time conditions for biofilm forming on CLs Comparison of the *S. aureus*, *P. aeruginosa* and *C. albicans* biofilms formed in artificial tear solution A: with or without shaking, B: at 24 h or The X- and Y-axis represents CL types, and logarithmic microorganisms’ survival in biofilm, respectively.

10.7717/peerj.9419/supp-3 Figure S3 Activities of MPSs against standard and clinical *S. aureus* biofilms on CLs MPSs’ in vitro activities observed by time-kill determinations against biofilms of standard (left) and clinical (right) * S. aureus * strains on Softens 38 (A and B), and Acuve 2 (C and D) CLs. The X- and Y-axis represents time, and logarithmic * S. aureus* survival in biofilm, respectively. cfu: colony-forming unit, Error bars indicate the standard deviations between repeated tests. Control: * S. aureus* biofilms without any antimicrobial treatment.

10.7717/peerj.9419/supp-4 Figure S4 Activities of MPSs against standard and clinical *P. aeruginosa* biofilms on CLs MPSs’ in vitro activities observed by time-kill determinations against biofilms of standard (left) and clinical (right) *P. aeruginosa* strains on Softens 38 (A and B), and Acuve 2 (C and D) CLs. The X- and Y-axis represents time, and logarithmic *P. aeruginosa* survival in biofilm, respectively. cfu: colony-forming unit, Error bars indicate the standard deviations between repeated tests. Control: *P. aeruginosa* biofilms without any antimicrobial treatment.

10.7717/peerj.9419/supp-5 Figure S5 Activities of MPSs against standard and clinical *C. albicans* biofilms on CLs MPSs’ in vitro activities observed by time-kill determinations against biofilms of standard (left) and clinical (right) *C. albicans* strains on Softens 38 (A and B), and Acuve 2 (C and D) CLs. The X- and Y-axis represents time, and logarithmic *C. albicans* survival in biofilm, respectively. cfu: colony-forming unit, Error bars indicate the standard deviations between repeated tests. Control: * C. albicans* biofilms without any antimicrobial treatment.

10.7717/peerj.9419/supp-6 Supplemental Information 1 The numbers of survival microorganisms counts: CL graphics’ log cfu/ml values

## Additional Information and Declarations

### Data Availability

The following information was supplied regarding data availability: The raw data tables are available as a Supplemental File.
