# Supplementary figures and images for: Biofilm modelling on the contact lenses and comparison of the in vitro activities of multipurpose lens solutions and antibiotics
Source: PeerJ. 2020 Jun 24;8:e9419. doi: 10.7717/peerj.9419 (PMC7320721; doi:10.7717/peerj.9419)

A

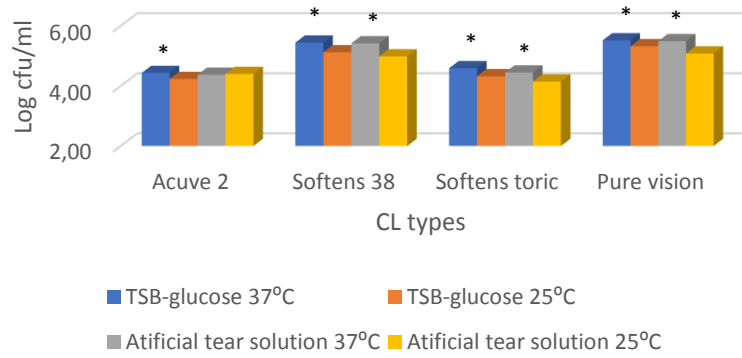

B

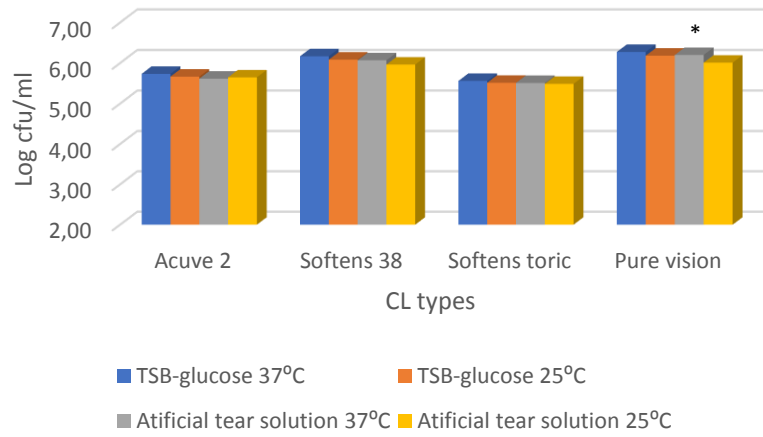

C

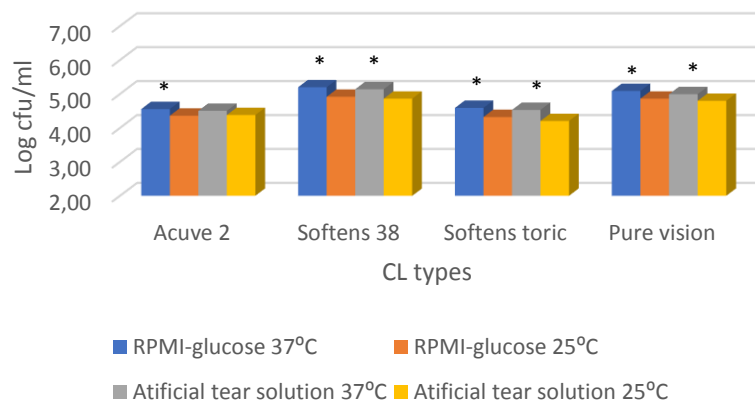

Supplement: Figure S1 — Comparison of the biofilms formed in different media and temperature conditions at 24 h for A: S. aureus and B: P. aeruginosa and 48 h for C: C. albicans standard strains. The X- and Y-axis represents CL types, and logarithmic microorganisms’ survival in biofilm, respectively. [file peerj-08-9419-s001.pdf]

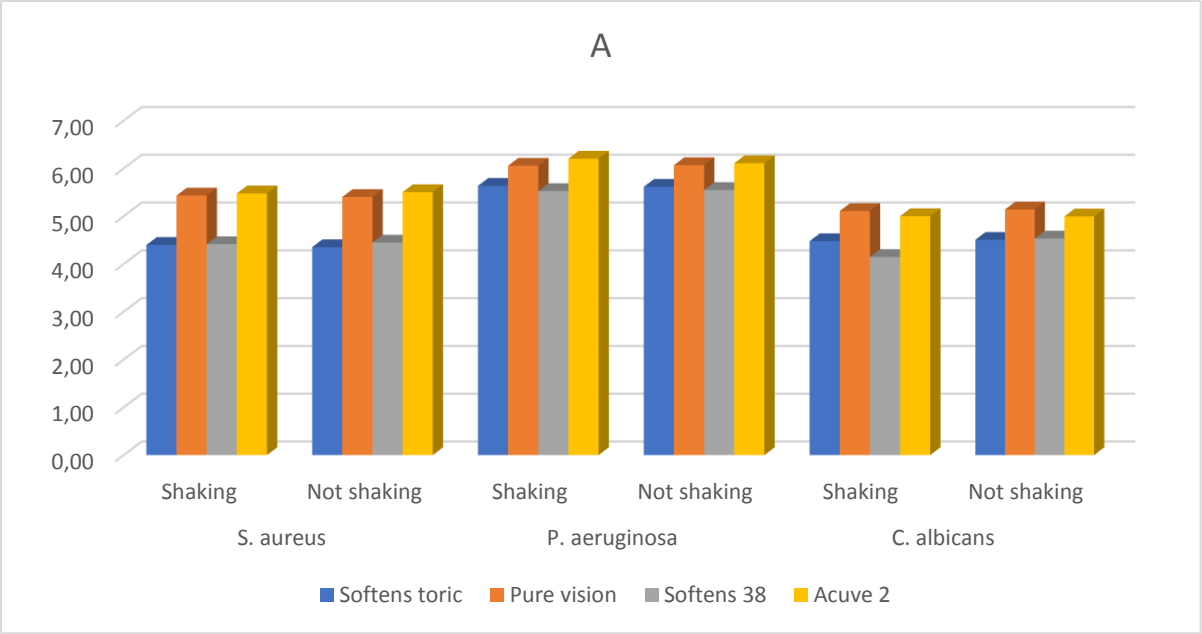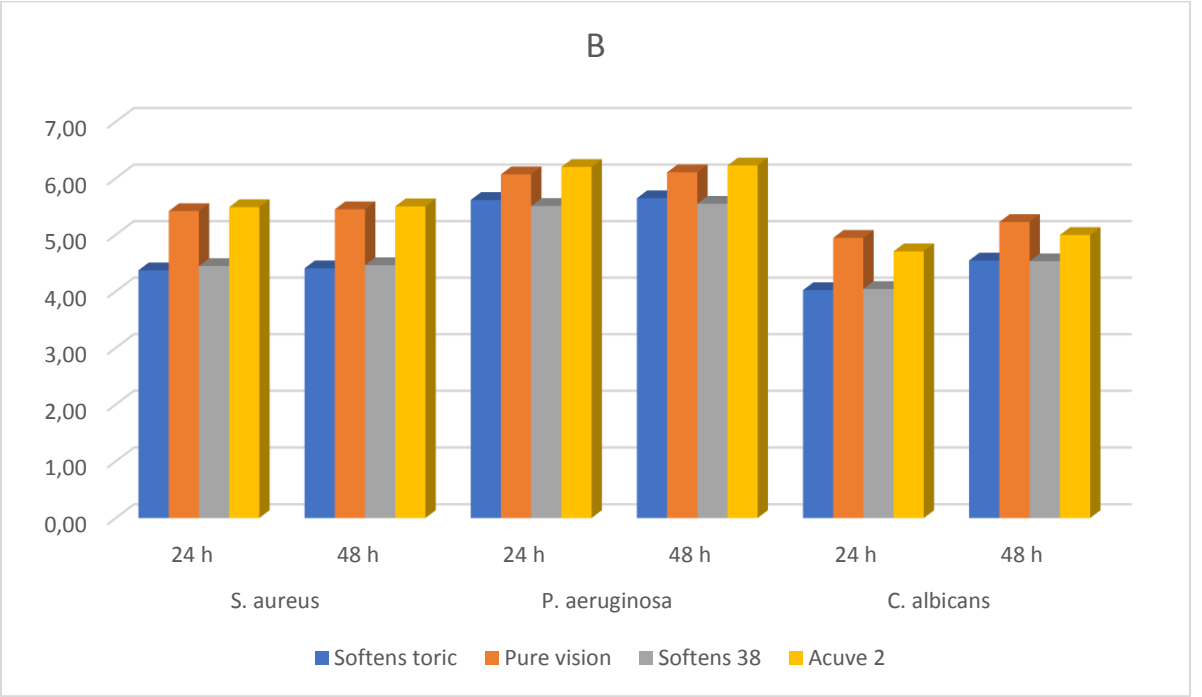

Supplement: Figure S2 — Comparison of the S. aureus, P. aeruginosa and C. albicans biofilms formed in artificial tear solution A: with or without shaking, B: at 24 h or The X- and Y-axis represents CL types, and logarithmic microorganisms’ survival in biofilm, respectively. [file peerj-08-9419-s002.pdf]

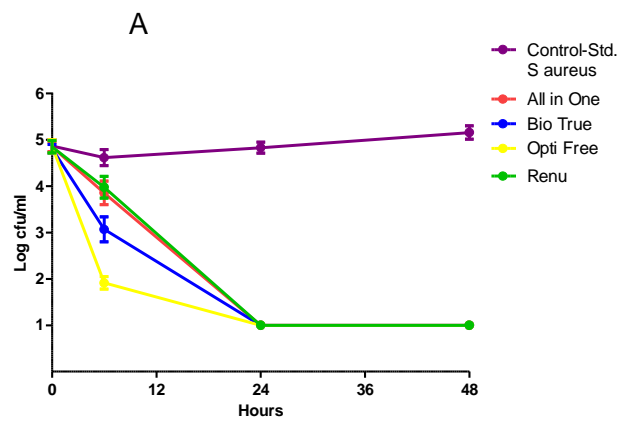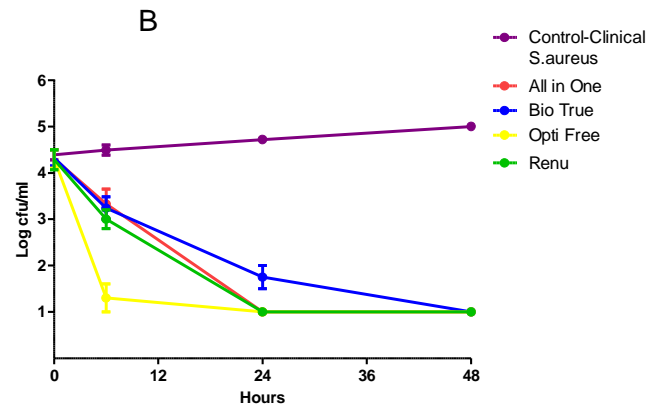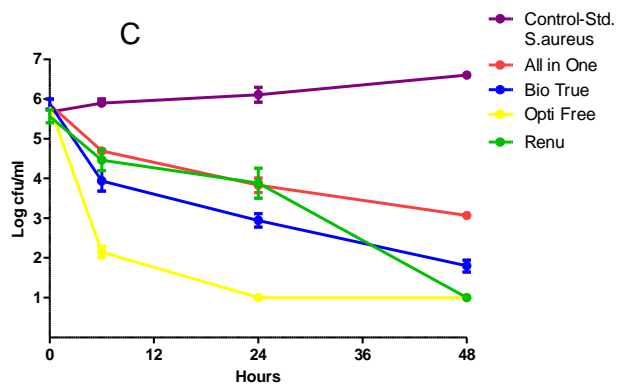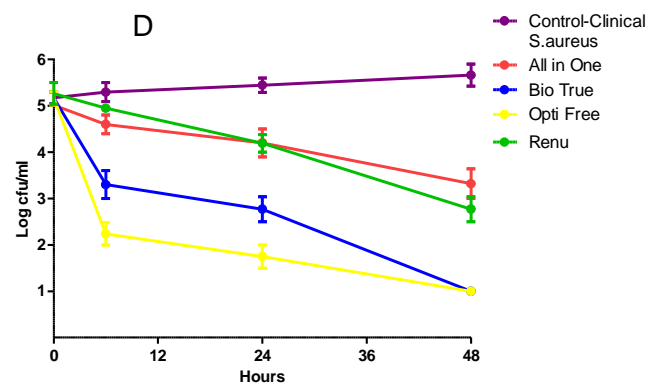

Supplement: Figure S3 — MPSs’ in vitro activities observed by time-kill determinations against biofilms of standard (left) and clinical (right) S. aureus strains on Softens 38 (A and B), and Acuve 2 (C and D) CLs. The X- and Y-axis represents time, and logarithmic S. aureus survival in biofilm, respectively. cfu: colony-forming unit, Error bars indicate the standard deviations between repeated tests. Control: S. aureus biofilms without any antimicrobial treatment. [file peerj-08-9419-s003.pdf]

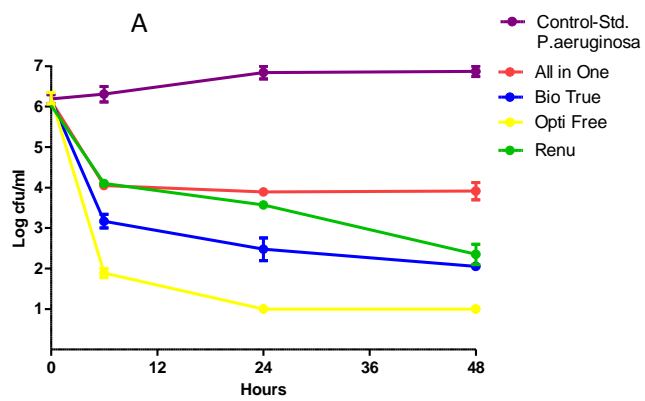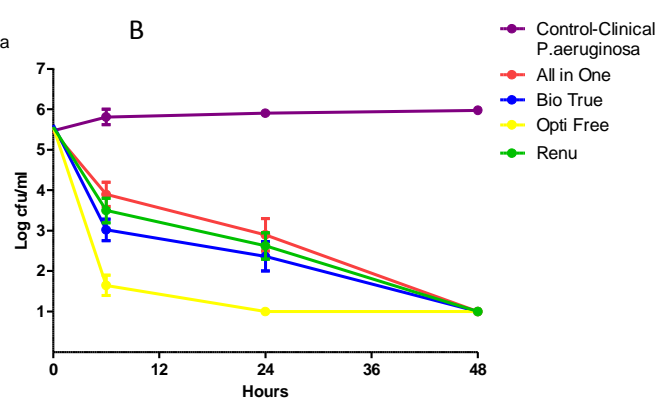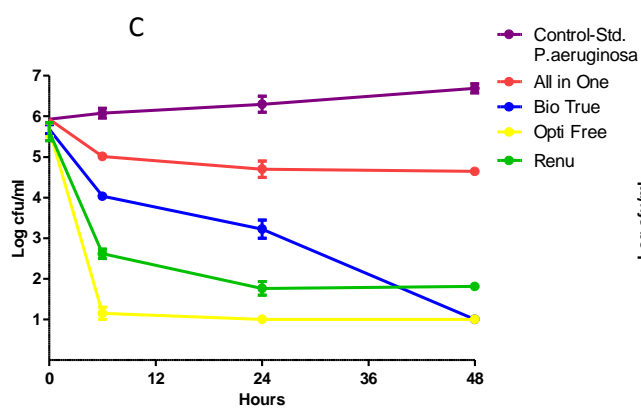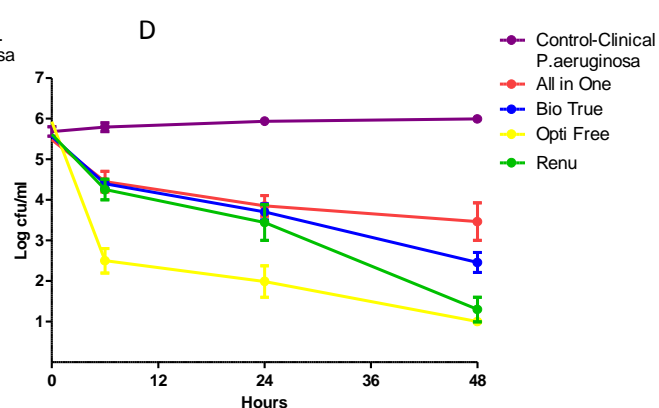

Supplement: Figure S4 — MPSs’ in vitro activities observed by time-kill determinations against biofilms of standard (left) and clinical (right) P. aeruginosa strains on Softens 38 (A and B), and Acuve 2 (C and D) CLs. The X- and Y-axis represents time, and logarithmic P. aeruginosa survival in biofilm, respectively. cfu: colony-forming unit, Error bars indicate the standard deviations between repeated tests. Control: P. aeruginosa biofilms without any antimicrobial treatment. [file peerj-08-9419-s004.pdf]

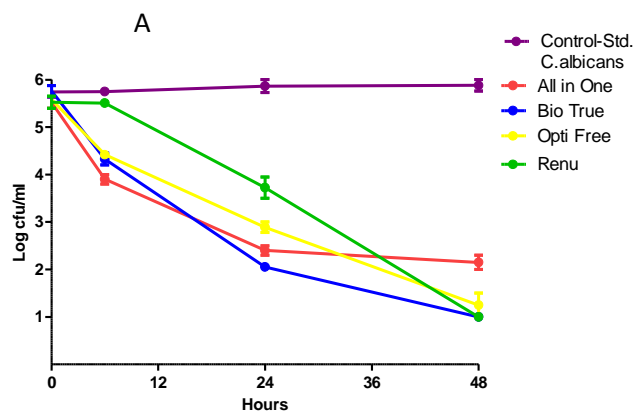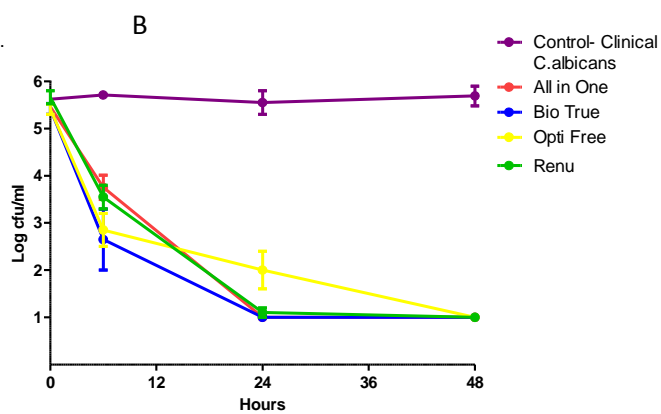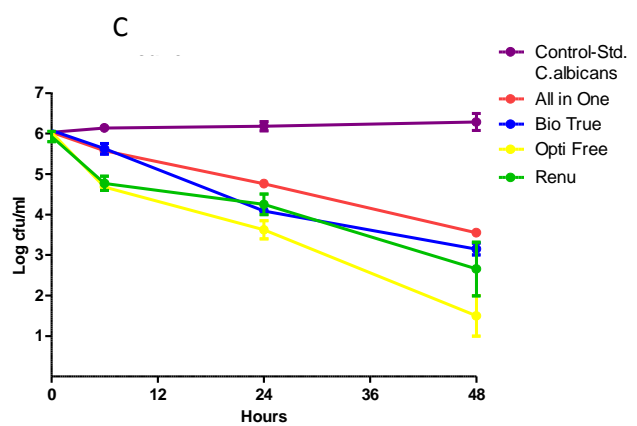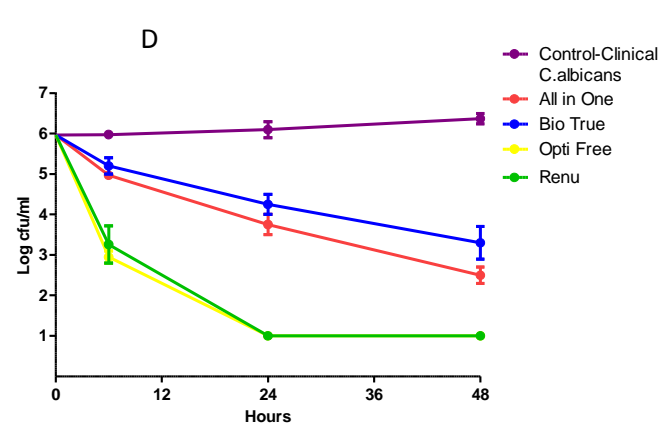

Supplement: Figure S5 — MPSs’ in vitro activities observed by time-kill determinations against biofilms of standard (left) and clinical (right) C. albicans strains on Softens 38 (A and B), and Acuve 2 (C and D) CLs. The X- and Y-axis represents time, and logarithmic C. albicans survival in biofilm, respectively. cfu: colony-forming unit, Error bars indicate the standard deviations between repeated tests. Control: C. albicans biofilms without any antimicrobial treatment. [file peerj-08-9419-s005.pdf]
